# An improved adaptive memetic differential evolution optimization algorithms for data clustering problems

**DOI:** 10.1371/journal.pone.0216906

**Published:** 2019-05-28

**Authors:** Hossam M. J. Mustafa, Masri Ayob, Mohd Zakree Ahmad Nazri, Graham Kendall

**Affiliations:** 1 Data Mining and Optimization Research Group, Center of Artificial Intelligence Technology, Faculty of Information Science and Technology, University Kebangsaan Malaysia, Bangi, Malaysia; 2 ASAP Research Group, University of Nottingham Malaysia, Malaysia; Northeast Electric Power University, CHINA

## Abstract

The performance of data clustering algorithms is mainly dependent on their ability to balance between the exploration and exploitation of the search process. Although some data clustering algorithms have achieved reasonable quality solutions for some datasets, their performance across real-life datasets could be improved. This paper proposes an adaptive memetic differential evolution optimisation algorithm (AMADE) for addressing data clustering problems. The memetic algorithm (MA) employs an adaptive differential evolution (DE) mutation strategy, which can offer superior mutation performance across many combinatorial and continuous problem domains. By hybridising an adaptive DE mutation operator with the MA, we propose that it can lead to faster convergence and better balance the exploration and exploitation of the search. We would also expect that the performance of AMADE to be better than MA and DE if executed separately. Our experimental results, based on several real-life benchmark datasets, shows that AMADE outperformed other compared clustering algorithms when compared using statistical analysis. We conclude that the hybridisation of MA and the adaptive DE is a suitable approach for addressing data clustering problems and can improve the balance between global exploration and local exploitation of the optimisation algorithm.

## Introduction

Data clustering is widely used in different applications to understand the structure of the data, to focus on a specific set of clusters for further analysis, and to detect the characteristics of each cluster. Data clustering has been developed and used as an essential tool for different disciplines, in areas such as Information Retrieval [1], the Internet of Things [2], Business [3], Medicine [4], and Image segmentation [5].

In recent times, clustering methods have been extensively studied [6–8]. The clustering methods can be classified based on the fitness function. The popular clustering methods are classified as partitioning clustering methods. The partitioning clustering methods attempt to divide the dataset into a set of disjoint clusters and try to optimise specific criterion function, which may emphasise the local structure of the data. The most popular partitioning clustering algorithms are k-means, k-medoids, expectation maximisation, clustering large applications and clustering large application based on randomised search [9].

The K-means algorithm is one of the popular for centre-based clustering [9], which is recognised as being simple and efficient. However, K-means can detect only well separated, compact or spherical clusters [10]. It is sensitive to noise due to the use of squared Euclidean distance, where any data object in the cluster can significantly influence the centre of clusters. The performance of K-means is highly sensitive to the selection of initial centres [11]. Improper initialisation may lead to empty clusters, weak convergence or a high possibility of getting trapped in a local optima [9]. Some researchers overcome these drawbacks by using meta-heuristics, such as Genetic algorithms [12], Particle Swarm Optimization [13], Ant Colony Optimization [14], Black Hole Algorithm [15], Gravitational Search Algorithm [16] and Krill Herd algorithm [17].

In the clustering problems, the balance between exploration and exploitation can affect the ability of the clustering algorithm to find good clusters among the datasets being used [18]. Some of the earlier proposed clustering algorithms, based on meta-heuristics, managed to find good clustering solutions for specific datasets. However, across all datasets, it was unable to find good results, or the results were not robust [7]. This might be due to the imbalance between exploration and exploitation of the meta-heuristic algorithm, which may lead to premature convergence or stagnation [19]. Some researchers have proposed a hybrid approach of a global search with a local search in order to achieve a better balance. The global search handles exploration, while exploitation is handled by the local search [20–23]. Memetic Algorithms (MAs) are one type of hybrid evolutionary algorithms that offer an efficient optimisation framework by combining perturbation mechanisms, local search strategies, population management [24] and learning strategies [25]. MAs can adopt the strength of other optimisation algorithms by combining them within the same framework, which can provide better performance and overcome the weakness of other algorithms. MAs comprise evolutionary phases that aid its success in complex optimisation problems [26–29]. More specifically, the mutation, the improvement and the restart phases are primarily responsible for the stability of a MAs performance [30,31].

The differential evolution (DE) algorithm can be hybridised with the MA in the mutation phase, where DE offers a superior mutation performance across many combinatorial and continuous domains’ problems [32,33]. However, the DE algorithm is subject to stagnation problems [34]. Many researchers tried to use the adaptation approach with the DE mutation operator, where two trends were mainly focusing on the control parameter adaptation strategy [35] and adaptive strategy control [36]. The importance of the mutation strategy can guide the search process to a global optimum [37]. Therefore, [38] proposed global and local neighbourhood-based mutation operators, where it can balance between the global and local search throughout the evolutionary processes. However, the mutation vectors require well-selected weights for the global and local strategy.

### Related work

Many researchers have used the nature-inspired algorithms to overcome the shortcomings of the K-means algorithm, to avoid premature convergence. For example, [39] proposed a Gravitational Search Algorithm (GSA) for solving data clustering algorithm. The candidate solutions are created randomly and interact with one solution via Newton’s gravity law to find optimal solutions in the problem space. Later, [40] proposed a heuristic algorithm based on the Black Hole phenomenon, where it has a simple structure, easy, and free from parameter tuning implementation.

A hybrid meta-heuristic algorithm is proposed by [41] by using Particle Swarm Optimisation and Magnetic Charge System Search algorithms for partitioning clustering problem. A dynamic shuffled differential evolution algorithm (DSDE) is proposed by [42]. The DSDE used the DE/best/1 mutation strategy and shuffled frog leaping algorithm to separate the population into two groups of the population during the evolving process.

In recent researches, authors presented data clustering algorithms by integrating the K-means data clustering algorithm with the population-based meta-heuristics algorithms, for example; Abdeyazdan presented an enhanced data clustering approach for that adopts the combination of the K-harmonic means algorithm (KHM) and a modified version of the Imperialist Competitive Algorithm (ICA) algorithm [43]. Gong et al. presented an improved Artificial Bee Colony clustering algorithm by enhancing the initial clustering centres selection [44]. Mustafi et al. presented an improved Genetic Algorithm (GA) data clustering algorithm to overcome the K-means clustering algorithm drawbacks [12]. Niu et al. proposed an integrated Particle Swarm Optimisers (PSOs) with the K-means algorithm [13]. Pandey et al. proposed Improved Cuckoo Search data clustering that adopts the K-means [45]. In the research of [46], the authors proposed an improved Tabu Search strategy that is integrated with the K-means clustering algorithm. More recently, The research of [19] combined the K-Harmonic Means (KHM) algorithm with PSO and an improved Cuckoo Search (ICS). They used ICS and PSO to avoid the problem of falling into the local optima.

Despite that the modified data clustering algorithms based on many evolutionary approached have better performance than other earlier algorithms, there still a problem with the weak convergence shortcoming in some evolutionary algorithms. More precisely, the exploitation and exploration balance of the evolutionary algorithms can be further improved. Therefore, in this work, we aim to improve the clustering algorithm based on an adaptive memetic differential evolution, named AMADE.

### Contribution of this paper

The main objective of this paper is to address the issues discussed above by proposing an adaptive memetic differential evolution for solving data clustering problems. Specifically, the significance of our contribution is three-fold.

1. We design an adaptive memetic differential evolution algorithm for the data clustering problem. The proposed algorithm data clustering algorithm used the approach of combining MA and DE in order to solve the data clustering problem.
2. We develop an adaptive DE Mutation phase with an adaptive mutation strategy that can be used to narrow the search process through the evolutionary steps generations to the nearest possible centroids.
3. We develop a local search algorithm utilising a neighbourhood selection heuristic that seeks better centroids based on the maximum and minimum values of each attribute centroid.

More specifically, the algorithm proposed an adaptive DE mutation operator that was combined with memetic algorithm evolutionary steps. The mutation operator strengthens the search capabilities of DE through proposed DE mutation strategy. Thus, the algorithm also introduced an adaptive strategy to avoid any stagnation problem. The DE Mutation phase employs an adaptive DE/current-to-best/1 mutation strategy, to speed up the convergence speed of differential evolution algorithm under the guidance of both current and the best individuals.

The memetic improvement phase included two steps: removing the duplicate solutions and local search using an improved neighbourhood search heuristic, the modification aimed to prevent the algorithm from falling into premature convergence. The hill-climbing local search algorithm is utilised as a local search algorithm to seek for better centroids by employing an improved neighbourhood selection heuristic with first improvement strategy. The neighbourhood selection heuristic seeks better centroids based on the maximum and minimum values of each attribute centroid. The restart phase was modified to replace the new partial population with good solution generate from the discrete differential algorithm, which can keep the diversity of the population as maximum as possible.

### Organization of This Paper

This research is organised as the following: Section 2 introduces the theoretical background and concepts such as standard MA and DE. In section 3, briefly explains the improved adaptive memetic DE. Section 4 presents the experimental results of the proposed algorithm. Finally, Section 5 provides the conclusion and future works.

## Background

This section discusses the fundamental aspects of clustering analysis problem, differential evolution (DE) and memetic algorithms, which have been used in the proposed data clustering algorithm. Thus, this section discusses the relevant population-based approaches in the data clustering.

### Cluster analysis

Data Clustering is a process of partitioning a set of *n* objects into some clusters *K*, based on a specific similarity measure. The *n* objects are represented by the set *X = {x*_*1*_, *x*_*2*_, *…*, *x*_*n*_*}*, the *K* clusters are denoted by *C = {C*_*1*_, *C*_*2*_, *…*, *C*_*K*_*}*, such that data objects in the same clusters are similar, and other data objects are dissimilar. In the data clustering problem, clusters must maintain the following three hard constraints [47]:

- Each cluster should consist of at least one object:
  $$C_{i}\neq \phi ,\forall \;i\in \{1,2,\ldots ,K\},$$ (1)
- Different clusters should not have objects in common:
  $$C_{i}\cap C_{j}=\phi ,\forall \;i\neq j\;and\;i,j\in \{1,2,\ldots ,K\},$$ (2)
- Every object must be attached to a cluster:
  $${⋃}_{i=1}^{k}C_{i}=X$$ (3)

The Data clustering problem can be represented the Eq (4):

$$\begin{array}{c} Optimize\\ \;C \end{array}f(X,C)$$ (4)

The *f(X*, *C)* is the fitness function to measure the quality of the partitions generated by the clustering method. Thus, the fitness function can be maximised or minimised depending on the similarity/dissimilarity measure used. Moreover, the fitness function should be defined for adequate partitioning. The intra-cluster Distance similarity/dissimilarity measure is one of the most popular internal metrics that is utilised to measure the quality of the clustering solution [7], as in the Eq (5):

$$f(O,C)=\sum_{l=1}^{k}\sum_{Oi\in Cl}^{n}d(O_{i},Z_{l})$$ (5)

The *d(O*_*i*_, *Z*_*l*_*)* represents the distance between the centre of cluster *Z*_*l*_ and data object O_i_. The Euclidean distance, as in Eq (6), is one of the most famous distance functions [7]. It can measure the distance between two objects (O_i_ and O_j_) inside the same cluster.

$$Euclidean\;distance\;d(O_{i},O_{j})=\sqrt{\sum_{m=1}^{d}{\left(O_{i}^{m}-O_{j}^{m}\right)}^{2}}$$ (6)

Furthermore, the centres *Z*_*l*_ is can determine the mean value for all cluster objects as in Eq (7), where the number of data objects in cluster *Z*_*l*_ is denoted by *n*_*l*._

$$Z_{l}=\frac{1}{n_{l}}\sum_{\forall O_{i}\in Z_{l}}\left(O_{i}\right)$$ (7)

### Differential evolution algorithm (DE)

DE algorithm is an effective meta-heuristic optimisation algorithm for solving continuous and combinatorial optimisation problems [48]. The algorithm starts with initialising a population. The individuals are chosen as parents for the mutation and crossover operators to generate trial offspring individuals. The mutation operation perturbed a base individual by a scaled difference vector, where the vector can consist of many random individuals selected from the population in order to produce a mutant individual. The comparison between offspring individual with the parent in fitness value will result in a new individual for the next generation. The evolution process will be terminated when satisfying a termination condition. Finally, in the last generation, the best individual will be the solution to the problem. The DE algorithm starts the evolutionary process by initialising the population with individuals in the solution space. In each generation, the individuals are selected as parents for the mutation and crossover in order to generate the trial offspring individuals. In the mutation phase, the individual is perturbed by a scaled differential vector that contains several individuals that are randomly selected in order to produce the mutant individual. The offspring individual is then compared with the parent using the fitness value, and the superior one is chosen as the new individual for the next generation. The evolutionary processes are terminated when the termination condition is satisfied, and the solution to the problem will be the best individual in the last generation.

The effectiveness of the DE in solving complicated optimisation problems depends mainly on choosing suitable mutation strategy and the related parameter values. Therefore, choosing suitable control parameter values for the DE algorithm is an essential task. Many researchers have been attracted to study the DE algorithm. For example, [35] proposed DE-PAS algorithm for selecting and incorporating a suitable adapting parameters scheme. [49] proposed a network intrusion detection based on efficient feature selection technique using decision tree algorithm and discretised differential evolution (DDE) from standard intrusion datasets. [50] presented a DE algorithm that can avoid premature convergence and improve the search quality. The population is grouped into many tribes and utilises an ensemble of different mutation and crossover strategies. They used an adaptive scheme to control the scaling factor and the crossover rate. [51] introduced a self-adaptive differential evolution algorithm (APDDE). The algorithm integrates the detecting values into two mutation strategies to produce the offspring population. [36] proposed a self-adaptive differential evolution algorithm with a hybrid mutation operator (SHDE) for parameters identification problem. In [52], researchers proposed a self-adaptive DE which can predict the control parameters based on the ensemble. [53] proposed a self-adaptive DE algorithm with discrete mutation control parameters (DMPSADE). Every individual contains its mutation control parameter, crossover control parameter and mutation strategy.

### Memetic algorithms

The Memetic Algorithms (MAs) are a meta-heuristic approach that combines the problem-specific solvers with evolutionary algorithms. The problem solvers can be implemented using exact methods, approximation algorithms or local search heuristics. The hybridisation aims to accelerate the discovery of good solutions or to find the solutions that are unreachable by evolutionary algorithms or the local search methods alone. MAs have been proven successful performance for a broad range of problem domains, such as wireless sensor networks [54], Machine learning algorithms [55], scheduling problems [56], routing problems [57] and bioinformatics [58]. MAs received many names throughout the literature. Some of the alternative names are hybrid GA, Baldwinian EA, Lamarckian EA, genetic local search algorithms [59]. The MAs can combine techniques and approach from many search techniques, and most distinguished approaches from local search methods and population-based search techniques. The basic memetic algorithms template include procedures:

#### The initialisation procedure

The initialisation procedure is responsible for creating solutions to the initial set of the population. The MAs seeks to create high-quality solutions to be in the starting point. The initialisation procedure can be done either using a local search procedure or a constructive heuristic to improve the random initial solutions.

#### The cooperate and improve procedures

The cooperate and Improve procedures typically rely on the selection of the solutions from the population and recombine them. Both procedures utilise the approach of a local search in the population.

#### The compete procedure

The Compete procedure is used in the reconstruction of the current population using the old and the new population. A steady-state replacement strategy is one of the most popular strategies that could be used when the fitness function suffers from complexity and time-consumption and could lead to faster convergence [59].

#### The restart procedure

The restart procedure is invoked whenever the population falls into a degenerate state. Typically, one of the strategies that could be used is to keep a part of the current population and generate the remaining part by new solutions. Another approach is to apply a heavy mutation operator; this could generate a population different from the current state in the search space.

#### Improved adaptive memetic differential evolution

This section discusses the detailed steps of the propose AMADE algorithm along with the solution representation.

### Solution representation

**Fig 1:**
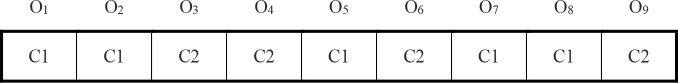
Example of a candidate solution represented by a label-based representation.

The optimal encoding aims to determine the data objects that belong to a particular cluster to perform optimal clustering analysis. A label-based one-dimensional array is used to represent the candidate solution in data clustering optimisation problem. Every solution representation is considered as a set of *N* data objects, where each cell represents a cluster number associated with that object. Fig 1 presents a candidate solution example for a problem with nine data objects and two clusters, In this example: objects *O*_*1*,_ *O*_*2*,_ *O*_*5*,_ *O*_*7*_ and *O*_*8*_ is attached with clusters 1, and *O*_*3*,_ *O*_*4*,_ *O*_*6*_ and *O*_*9*_ is attached with clusters 2.

**Fig 2:**
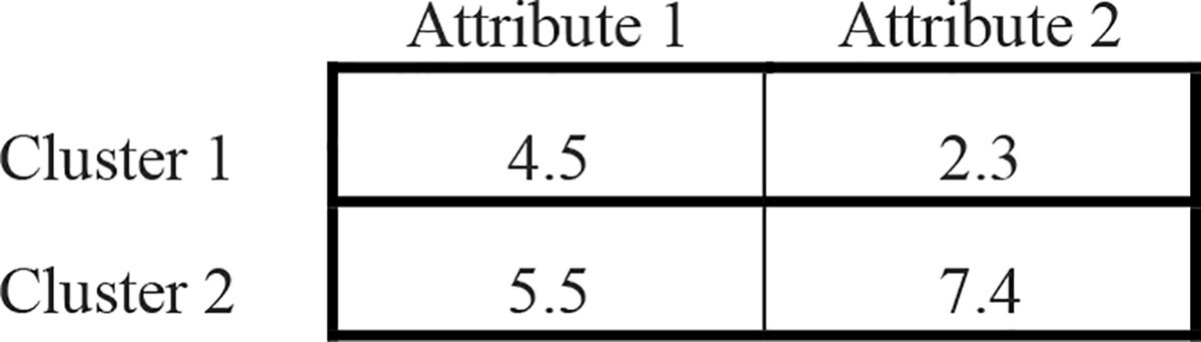
Cluster centroid-based representation of the solutions.

Additionally, a centroid-based representation that consists of a two-dimensional matrix is used to keep track of the positions of the cluster centroid and to be used by the local search. The matrix consists of *K* rows and *D* column, where *K* is the total number of the clusters and *D* is the total number of the attributes in the dataset. For example, in Fig 2, the dataset contains two clusters and two attributes; then the position of first cluster centroids is 4.5, 2.3, and the position of the second cluster centroids is 5.5, 7.4.

### Constraint handling

The solution representation of the proposed AMADE guarantees that each data object is associated only with one cluster. An additional soft constraint is formed to prevent any duplicate solutions in the population in the improvement phase. Moreover, any possible duplicate solutions can lead through the evolutionary processes to premature stagnation. The duplicate solution is handled in the improvement phase by generating solutions randomly.

### The AMADE proposed approach

**Fig 3:**
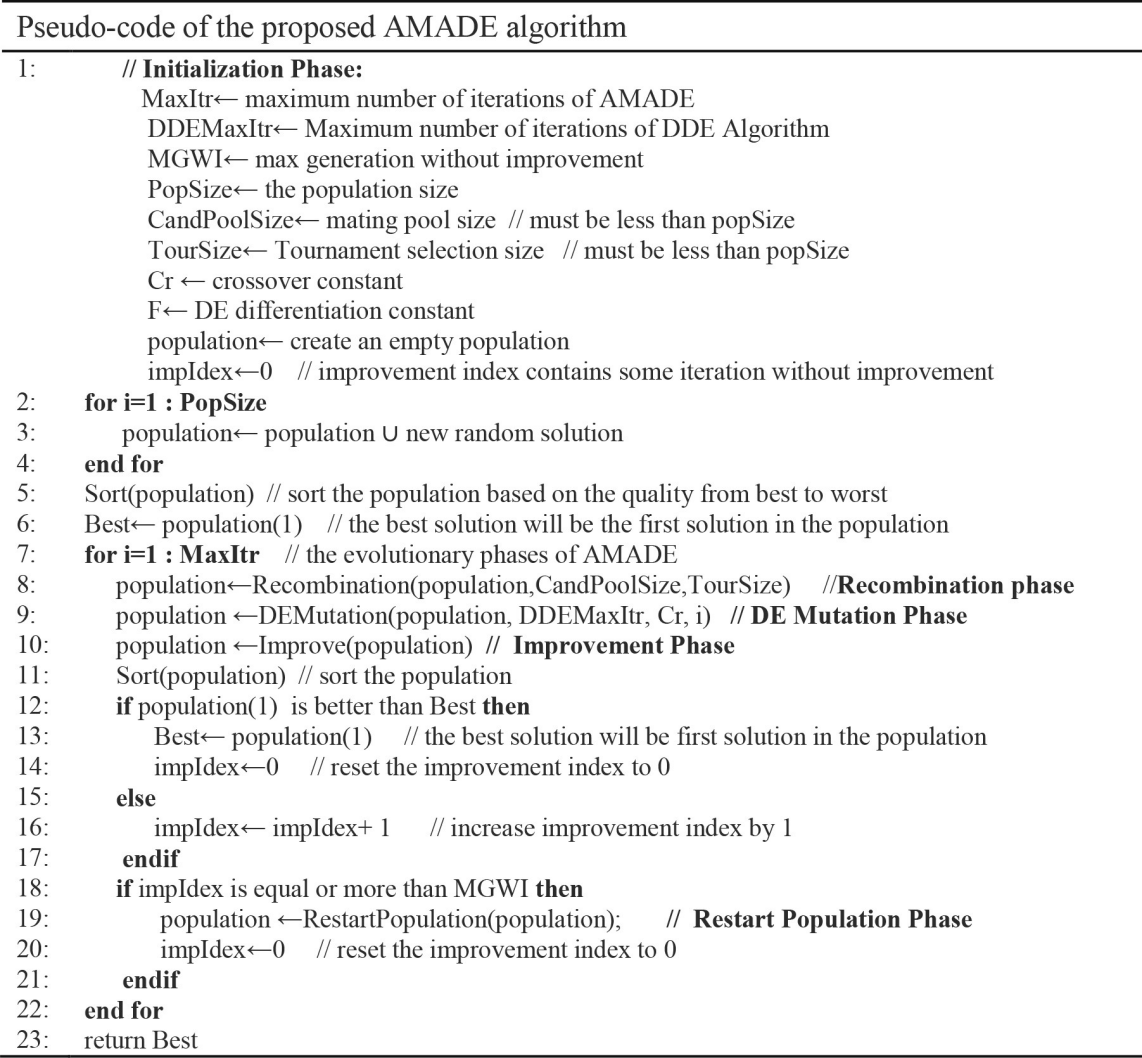
Pseudo-code of the proposed AMADE algorithm.

In AMADE, the DE mutation operator with an adaptive strategy *DE/current-to-best/1* has combined with the memetic algorithm evolutionary steps; this aims to have faster convergence speed faster by the best individual’s guidance. The new individuals are compared with the target vector, which can improve the guidance of the population evolution. However, AMADE may suffer from premature convergence. To this end, the restart phase can prevent falling into premature convergence by reconstructing the population diversity by generating new solutions in the population. The improvement phase plays a key role in finding better solutions, which can also improve the quality of the solution using a proposed improvement heuristic. The pseudo-code for the proposed AMADE algorithm is shown in Fig 3.

### The population initialisation phase

For keeping better diversity of the population, a random constructive method is used. The initial solutions for the proposed AMADE are randomly generated. The data points of the dataset are randomly grouped into *K* clusters; all centroids of each cluster are calculated using Eq (7), where *K* is the total number of clusters. These two steps are repeated *N* times to generate *N* random solutions, where *N* is the population size parameter value of the AMADE algorithm.

### The recombination phase

**Fig 4:**
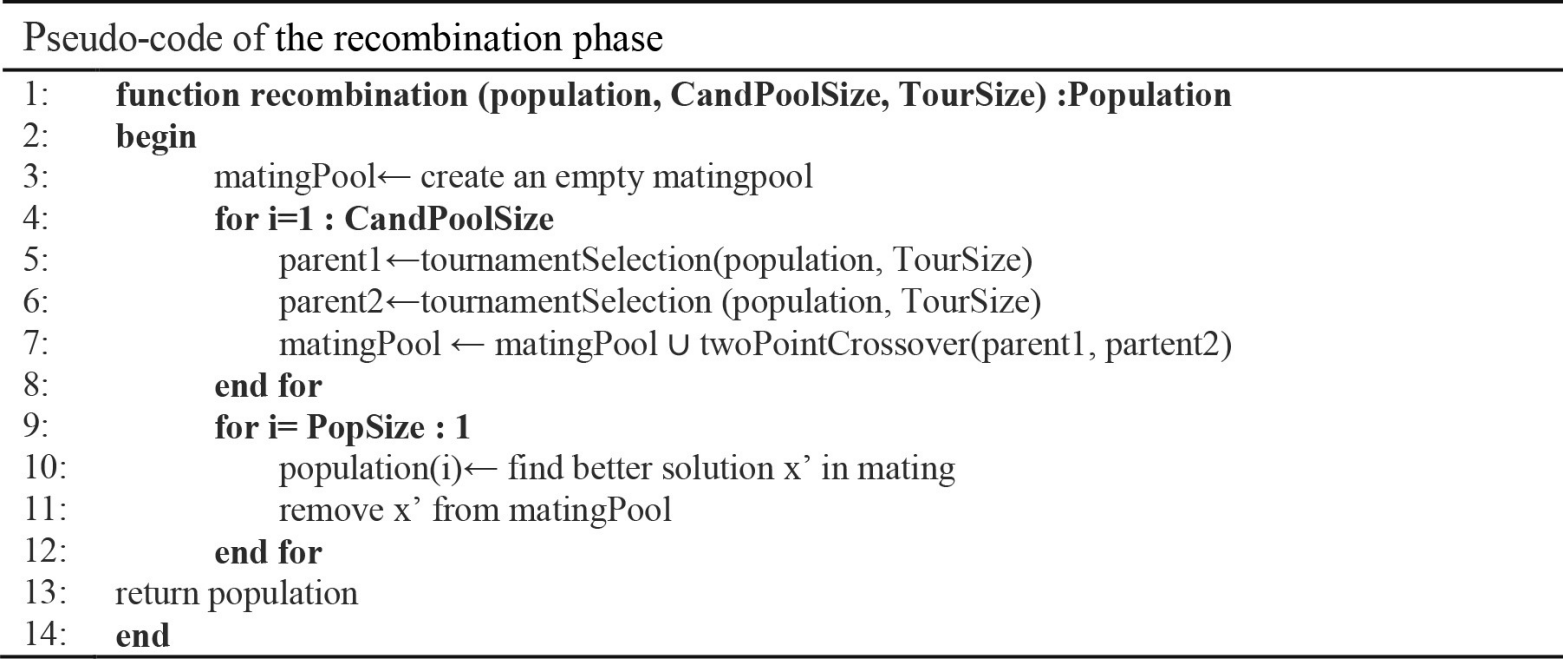
Pseudo-code of the recombination phase algorithm.

The recombination phase employs the mating pool approach [60] in evolutionary computation with *CandPoolSize* size. The tournament selection operators, with selection size *TourSize* [61] are applied to the entire population then placed into the mating pool. Thus, a two-point crossover operator [62] is then applied to the selected parents. At the end of the recombination phase, the mating pool is combined with the population by replacing some worst individuals with the new better individuals in the mating pool. The pseudo-code of the recombination phase is shown in Fig 4.

**Fig 5:**
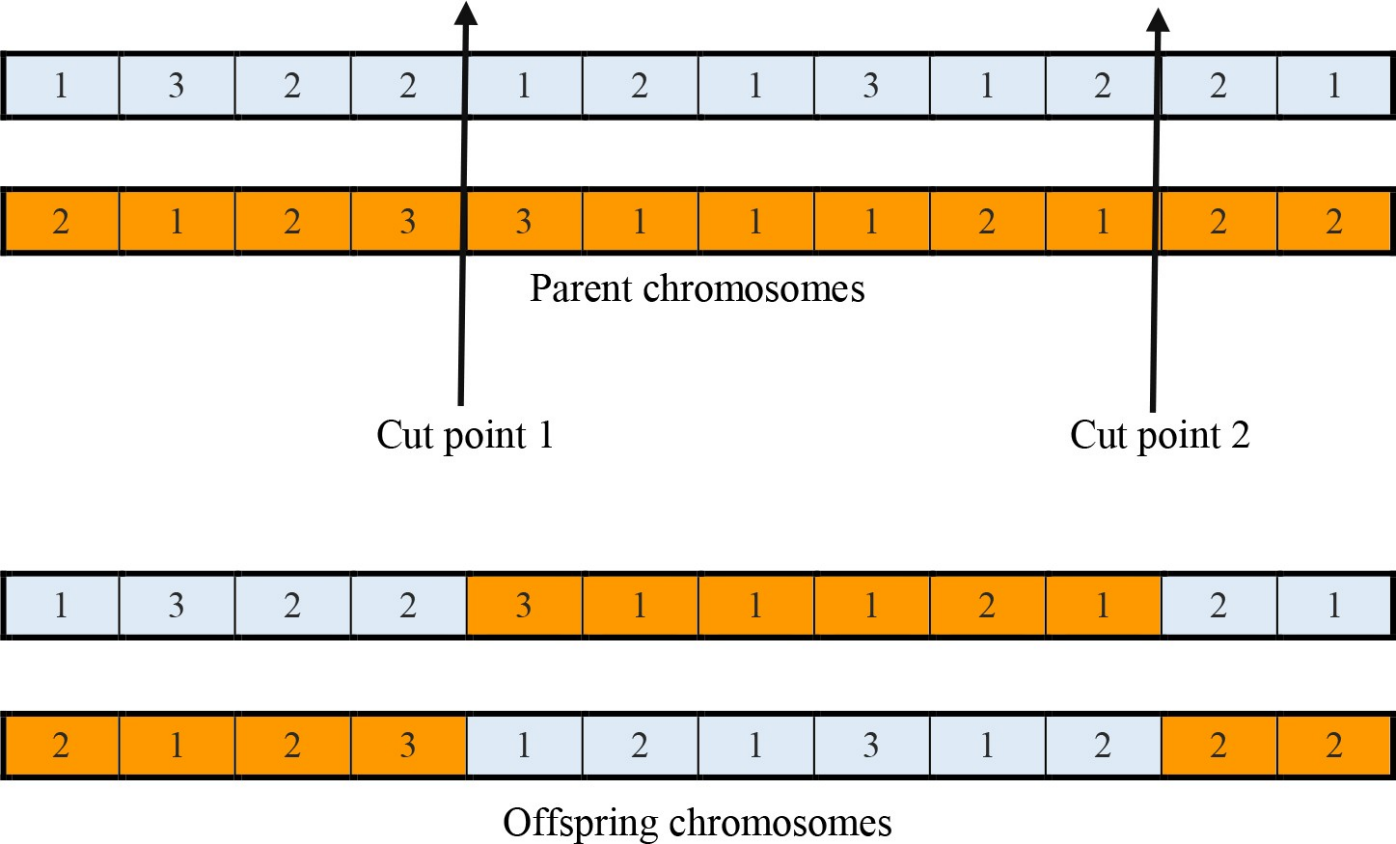
Example of two-point crossover with label-based solution representation for a clustering solution using of twelve data.

Fig 5 demonstrates an example of the two-point crossover with label-based solution representation chromosome with size *N* = 12 genes. The chosen cut points are randomly selected, and cut point 1 should be less than cut point 2.

### The DE Mutation phase

**Fig 6:**
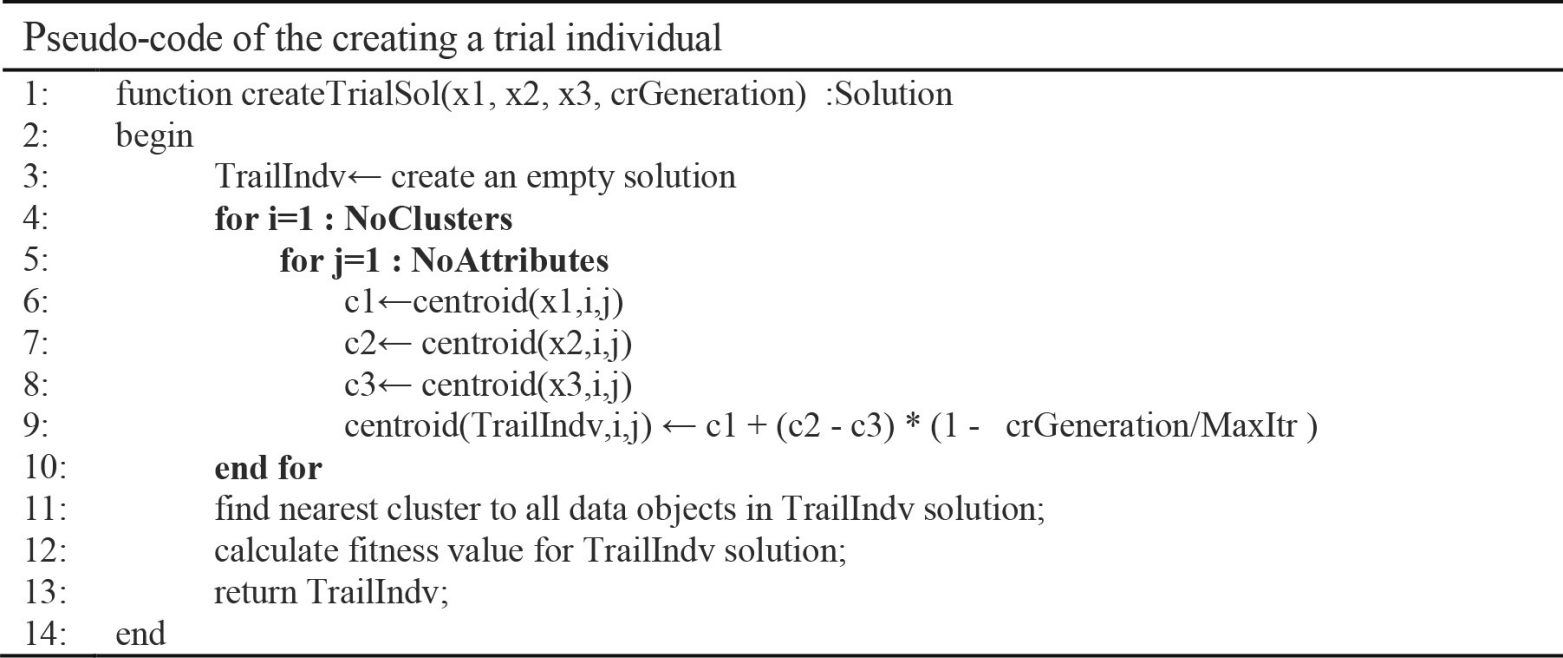
Pseudo-code of creating the trial individual algorithm.

The DE Mutation phase employs an adaptive *DE/current-to-best/1* mutation strategy, to speed up the convergence speed of DE algorithm under the guidance of both current and the best individuals. The cluster centroids are modified by the mutation phase to achieve better cluster solution, as shown in Fig 6. This is performed by using Eq (8). Where *C*_*current*_ is the current individual centroid, *C*_*best*_ is the best individual centroid, *C*_*rand*_ is a random individual centroid, *CurrIteration* is the current iteration in AMADE algorithm, and *MaxIterations* is the maximum number of iterations of AMADE.

$$C_{i}=C_{current}+\left((C_{best}-C_{rand})\times (1-\frac{CurrIteration}{MaxIterations})\right)$$ (8)

Such adaptive strategy will narrow the search process through the evolutionary steps generations to the nearest possible centroids. At the same phase, the data objects are rearranged to the closest clusters after modifying the centroids of the clusters. The new produced individual is immediately compared with the target vector in a current population, and the better individual could be retained.

**Fig 7:**
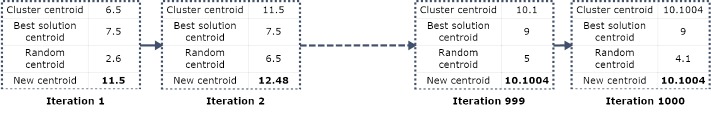
Example of the adaptive DE strategy performed on a cluster centroid of value 6.5 throughout 1000 iterations.

In order to demonstrate the effectiveness of the adaptive DE strategy, Fig 7 shows an example of a cluster centroid of value 6.5 that is adjusted throughout 1000 iterations of the AMADE algorithm. The adaptive DE strategy provides more exploration capabilities to cluster centroid, which in the first iteration is 6.5 and is adjusted to the new centroid that is 11.5. As the algorithm reaches the maximum number of iterations, the DE strategy produces more exploitation capability to the current centroids, which in the iteration 999 is 10.1 and the new centroid is 10.1004.

### The improvement phase

**Fig 8:**
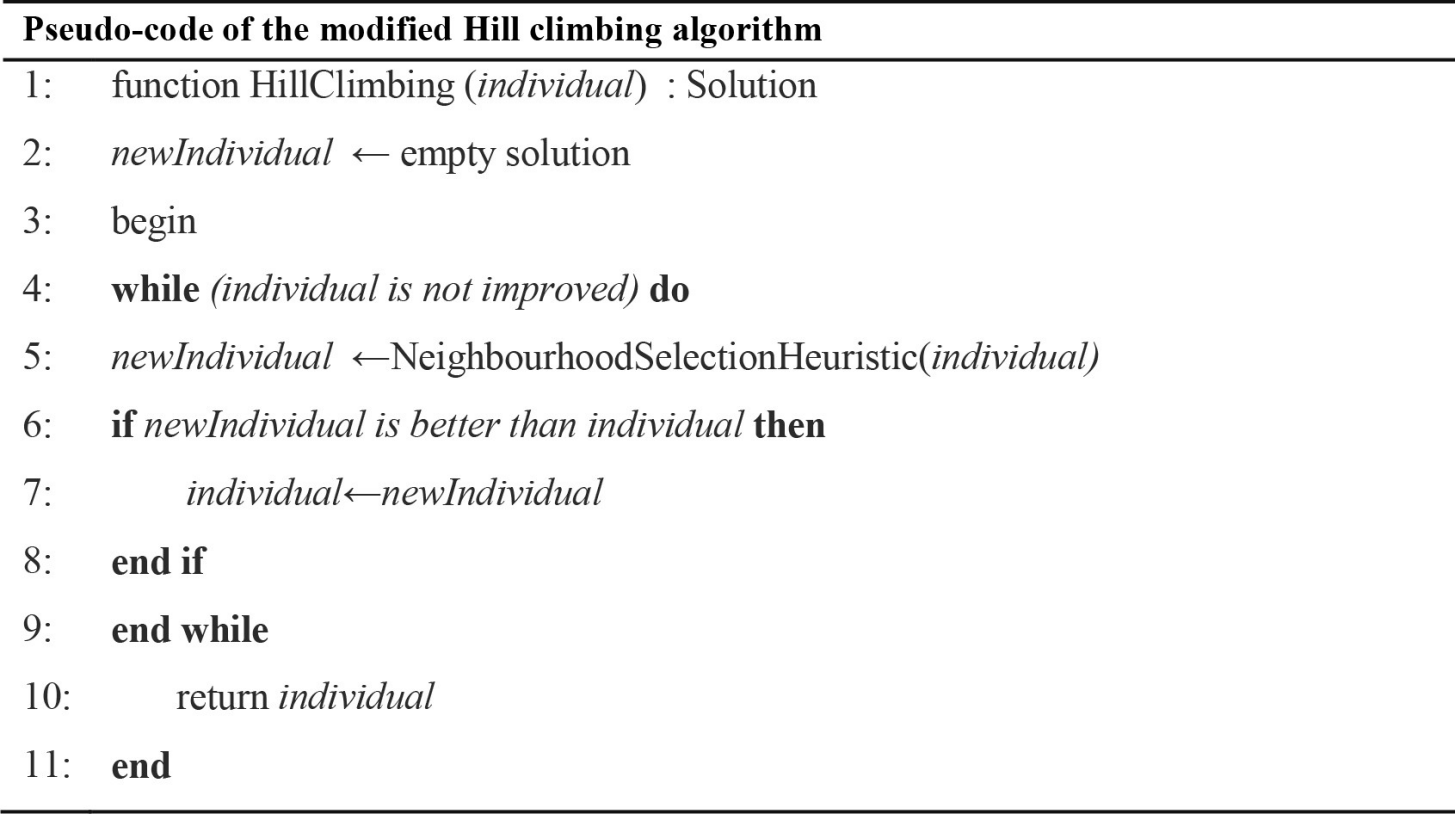
Pseudo-code of the modified Hill climbing algorithm.

The improvements phase consists of two solution quality improvement steps: the clear duplicate solutions step and the local search step. In the clear duplicate step, the algorithm ensures that the population retains better solution diversity in order to avoid any premature stagnation. At the second step, a hill-climbing local search [63] is employed on the centroid-based presentation by changing the current centroid with better cluster centroids. The hill-climbing local search algorithm, as shown in Fig 8, seeks better centroids by utilising the neighbourhood selection heuristic with a first improvement strategy [64]. The algorithm terminates when the current solution is improved.

**Fig 9:**
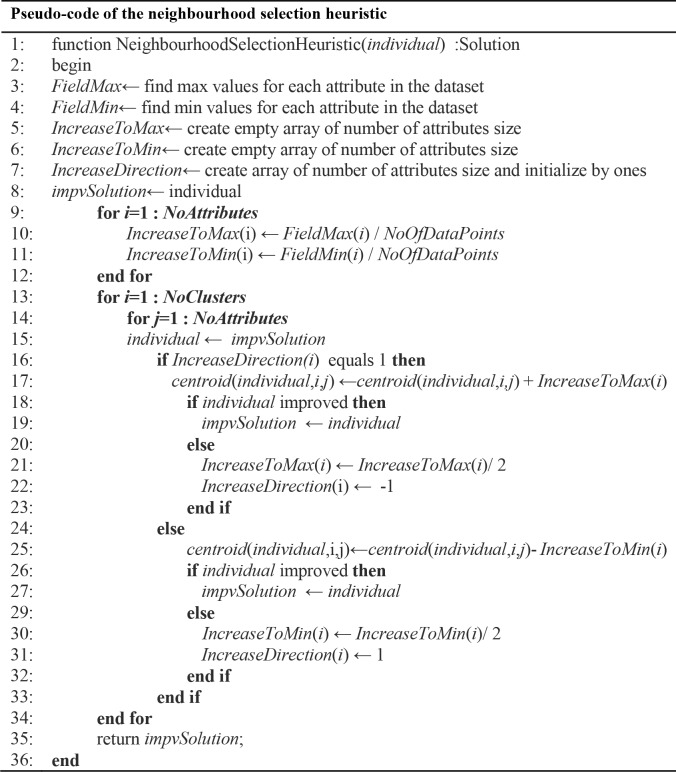
Pseudo-code of the neighbourhood selection heuristic algorithm.

**Fig 10:**
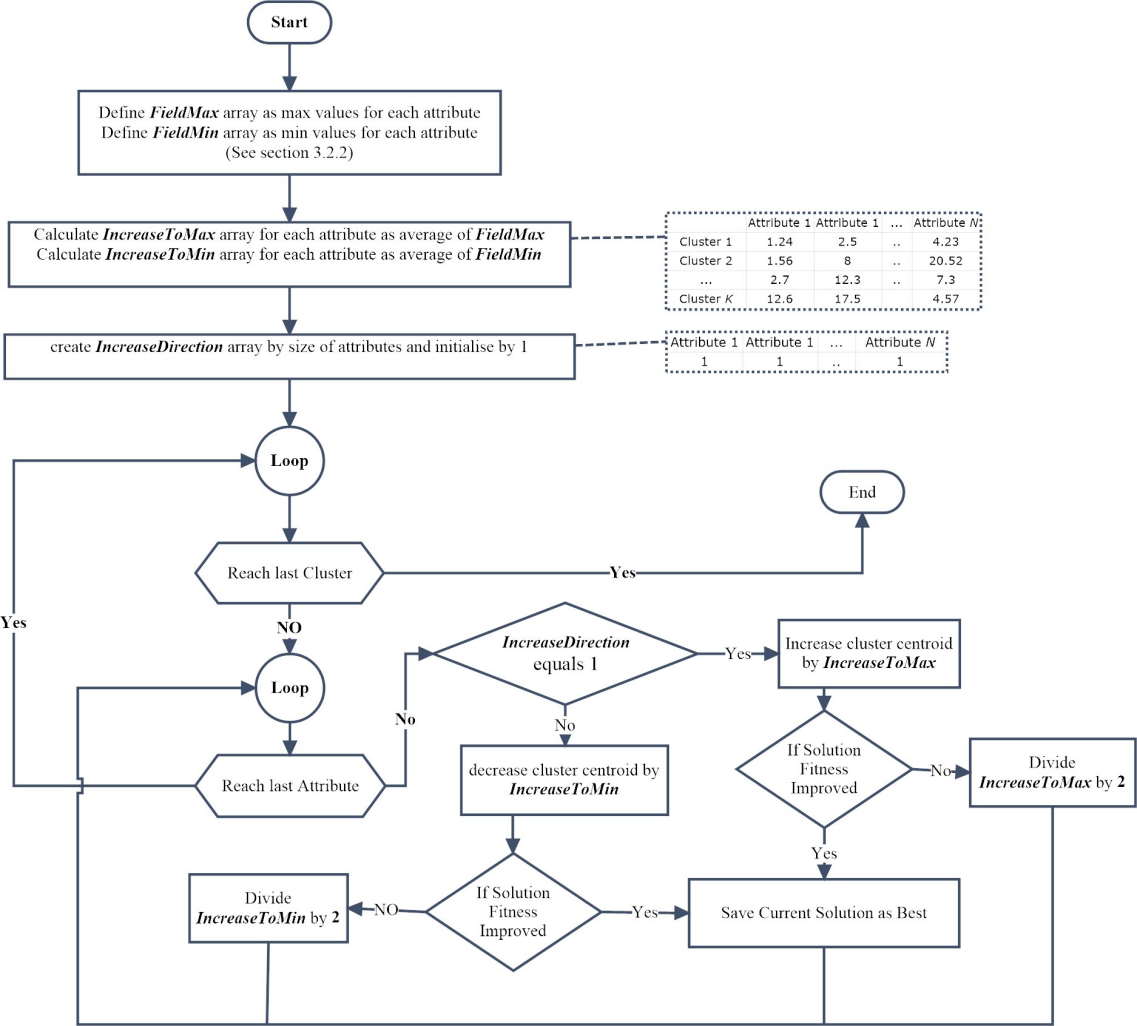
Flowchart of the neighbourhood selection heuristic.

The neighbourhood selection heuristic, as shown as pseudo-code in Fig 9 and a flowchart in Fig 10, seeks better centroids based on the maximum and minimum values of each field’s centroid. The heuristic increases the centroid value with an increment step value until finding better centroid. Otherwise, the algorithm will change the search direction decreasingly to the minimum value of centroid.

### The restart population phase

**Fig 11:**
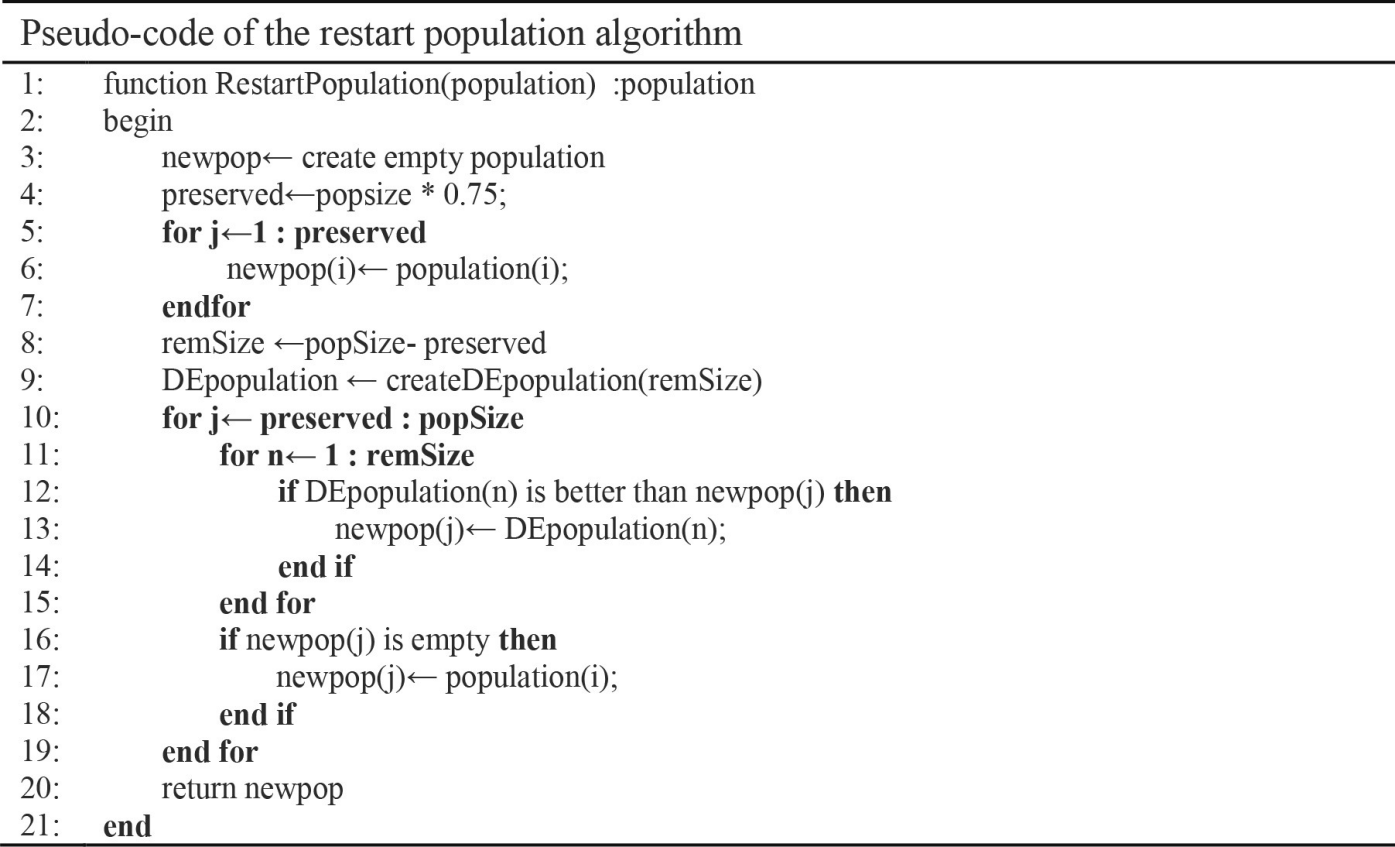
Pseudo-code of the restart population algorithm.

Once the population is having a state of degeneration, the restart procedure is employed immediately [59]. The restart strategy keeps part of the population and excludes the remaining individuals by generating new solutions. As shown Fig 11, AMADE keeps 75% of the population for the next evolutionary steps, while the remaining population is generated using a DE algorithm based on mutation strategy *DE/rand/1* and the minimal number of generations, which can produce a new population with better diversity and good quality solutions.

$$G_{i}=(G_{rand1}+F.(G_{rand3}-G_{rand3}))\;mod\;NoClusters$$ (9)

**Fig 12:**
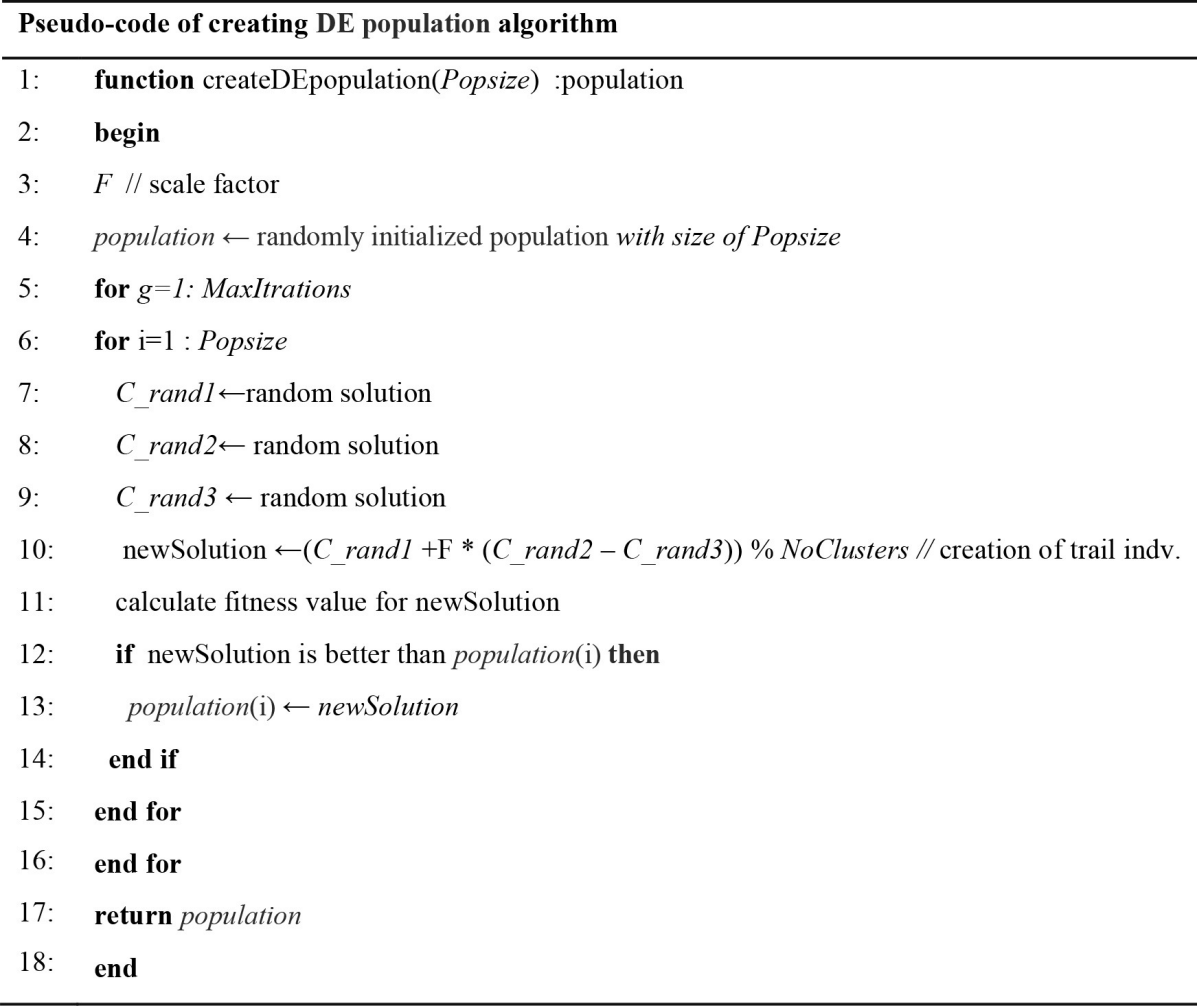
Pseudo-code of creating DE population algorithm.

The DE algorithm, shown in Fig 12, is applied to the solution representation with a discrete mutation operator. Each genome in the new chromosome is calculated using Eq (9). Where *G*_*rand1*_, *G*_*rand2*_, *G*_*rand3*_ is the gene in the chromosome of randomly selected individuals. The modulus is used to ensure that the result of the equation within the number of clusters in the dataset.

## Experimental setup and results

### Experimental setup

The performance of the proposed AMADE clustering method are investigated based on six real data datasets from the UCI repository of the machine learning databases with a variety of complexity [65], which can be download at http://archive.ics.uci.edu/ml/index.php. The datasets that been used are Wisconsin Breast Cancer, Vowel, Wine, Iris, Contraceptive Method Choice (CMC) and Glass, as shown in Table 1. The datasets also include different complexity levels and classified from 1 to 10 levels based on the number of instances and attributes [66], where level 1 is the lowest complexity level, and level 10 represents the highest complexity level.

**Table 1:** The characteristics of the UCI repository datasets used in the experiments of AMADE algorithm.

Dataset	Number of clusters	Number of features	Number of data objects	Description	Complexity levels
Vowel	6	3	871	Indian Telugu vowel	4
Iris	3	4	150	Fisher’s iris data	3
Cancer	2	9	683	Wisconsin breast cancer	3
CMC	3	9	1473	Contraceptive method choice	6
Glass	6	9	214	Glass identification data	3
Wine	3	13	178	Wine data	4

In order to evaluate the effectiveness of the proposed Memetic DE algorithm with proposed evolutionary phases, the AMADE performance is first compared with DE [48] with *DE/best/1/bin* strategy, Hybrid DE with *DE/best/1/bin* strategy, GA [67] and hybrid GA algorithms, where all algorithms are applied with the same experimental setup and local search heuristic. These algorithms have the same evolutionary phases of AMADA except restart phase. The selection of these algorithms is essential to show the strength of the combination of such algorithms in MA besides the proposed adaptive mutation operator and the modified restart phase. Moreover, for further testify the performance, the AMADE is compared with recent data clustering algorithms in the literature, including K-means [9], black hole [40], age-based particle swarm optimisation [68], dynamic shuffled differential evolution algorithm [42], the krill herd algorithm [69] and hybrid ICMPKHM [19].

The algorithm's performance is evaluated using the following criteria:

- The intra-cluster distances: is an internal quality measure that measures the distance between all objects in the cluster and its centre, as defined in Eq (5). The purpose of the data clustering algorithm is to minimise the sum of intra-cluster distances which can lead to high clustering quality. The intra-cluster distance value is given as best (minimum intra-cluster distance), the average value and worst (maximum intra-cluster distance) value of objective function value among entire runs.
- The F-measure: is an external measure that compares the ground truth with the obtained clusters to calculate the similarity between them. The high percentage of the F-measure value indicates a better clustering quality. The precision and recall of cluster *S*_j_, and class *R*_i_, *i*, *j* = 1, 2, …, *k* is shown in Eq (10) and Eq (11), Where |*R*_*i*_*|* is the number of objects in class *R*_*i*,_ and |*S*_*j*_| is the number of data objects in cluster S_*j*_, and *L*_*ij*_ is the number of data objects of class *R*_*i*_ in cluster *S*_*j*_. The *F-measure* of a class *R*_*i*_ is defined in Eq (12). The overall F-measure is computed as the weighted average of all classes is given in Eq (13).

$$precision\;(R_{i},S_{j})=\frac{L_{ij}}{\left|S_{j}\right|}$$ (10)

$$recall\;(R_{i},S_{j})=\frac{L_{ij}}{\left|R_{j}\right|}$$ (11)

$$F\;(R_{i})=\frac{2\times precision\;(R_{i},S_{j})\times recall\;(R_{i},S_{j})}{precision\;(R_{i},S_{j})+recall\;(R_{i},S_{j})}$$ (12)

$$F-measure(k)=\frac{\sum_{i=0}^{k-1}\left(|R_{i}|\times F(R_{i})\right)}{\sum_{i=0}^{k-1}\left|R_{i}\right|}$$ (13)

- The accuracy: is an external measure indicates the proportionate number of data objects that correctly placed by the predictive model to match the class (ground truth) in the data, as shown in Eq (14):

$$Accuracy\;(k)=\frac{number\;of\;correct\;data\;objects\;identified}{total\;number\;of\;data\;Objects}$$ (14)

The parameter settings for the AMADE algorithm were independently tested on each of the six datasets for 31 times, the best, worst, average values, standard deviations and F-measure were computed. In AMADE, the maximum number of generations is set to 1000, and 100 to DE/rand/1 in population restart phase. Accordingly, *F* is set to 0.7 and *Cr* is set to 0.9.

**Fig 13:**
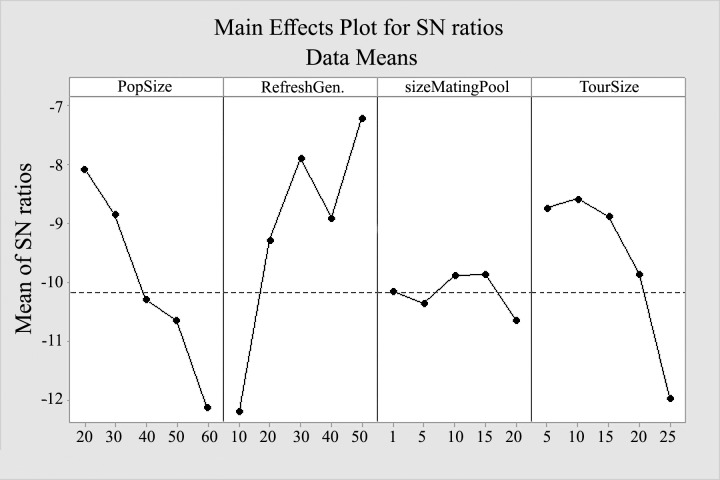
Graphical results of Taguchi method for AMADE algorithm.

A Taguchi method [70,71] for the design of the experiment has been used to identify the best values of the parameters for AMADE algorithm. Five levels were considered for each factor as shown in Table 2. AMADE algorithm run for 31 times for each factor at each level was employed, and the mean of signal-to-noise (SN*)* ratio plot each level of the factors are shown in Fig 13. The level with the maximum *SN* ratio is the optimum parameter determined by Taguchi method.

**Table 2:** The AMADE algorithm parameter levels.

Parameter	Definition	Level				
		1	2	3	4	5
PopSize	The population size	20	30	40	50	60
MGWI	max generation without improvement	10	20	30	40	50
CandPoolSize	recombination mating pool size	1	5	10	15	20
TourSize	tournament selection pressure	5	10	15	20	25

According to Fig 13, the optimum value for population size is set to 20, and max generation without improvement is set to 50. The recombination mating pool size is set to 10, and the tournament selection pressure is set to 10. Last but not least, All algorithms are implemented in Oracle Java 1.8 and were run on CPU Intel Core i7 (2.4GHz) personal computer that contains 8 GB of RAM.

### Experimental results and discussion

The objective function values comparison for the best solutions, average solutions and worst solutions, F-measure, standard deviation and execution time of solutions for 31 runs is shown in Table 3. Where Best, Mean and Worst are referred to the intra-cluster distances objective function values that were obtained out of 31 runs, where the smaller value is better, and the higher value of the F-measure is better. The results show that the proposed algorithm has a smaller best, average, worst and standard deviation compared with the other algorithms. For example, the Iris dataset results show that AMADE achieved 96.544 global optima whereas the best solutions of GA, DE, HyGA ad HyDE are 97.225, 97.101, 96.571 and 96.571. However, the worst, best, and average results of the solutions by HyGA and HyDE are close to AMADE on most of the datasets, but it did not perform well with the standard deviation and the results of worst solutions. Moreover, the results of F-measure of the proposed algorithm can be noticed as better than other algorithms in most datasets, except for the iris and cancer datasets which are similar to the global optimum.

**Table 3:** Comparison of intra-clusters distances among AMADE, HyDE, HyGA, DE and GA obtained from 31 runs.

Data Set	Criteria	GA	DE	HyGA	HyDE	AMADE
Iris	Best	97.225	97.101	96.571	96.571	**96.544**
	Mean	100.22	100.238	96.704	96.687	**96.549**
	Worst	106.63	121.42	97.082	96.851	**96.56**
	Std.	2.799	8.287	0.1332	0.0913	**0.004**
	F-measure	0.888	**0.901**	**0.901**	**0.901**	**0.901**
	Time (s)	0.063	**0.062**	3.311	2.908	2.442
Wine	Best	16555.679	16530.53	16295.932	16293.716	**16292.279**
	Mean	17469.554	16579.30	16307.626	16320.591	**16292.82**
	Worst	21381.732	18042.46	16375.151	16424.55	**16293.884**
	Std.	857.221	271.550	14.750	43.344	**0.395**
	F-measure	0.689	0.696	0.696	0.696	**0.708**
	Time (s)	**0.064**	0.105	15.170	6.229	5.279
Vowel	Best	213180.89	228726.3	149225.51	149216.30	**148967.54**
	Mean	338611.71	246848.6	150098.78	150537.95	**149228.50**
	Worst	414649.26	264116.7	151281.28	157436.32	**150121.94**
	Std.	58186.943	8847.791	**388.271**	1415.782	490.294
	F-measure	0.5731	0.549	0.645	0.549	**0.66209**
	Time (s)	**0.352**	0.825	25.901	15.333	14.166
CMC	Best	8232.03	7414.52	5534.209	5532.855	**5532.404**
	Mean	9913.725	8242.965	5538.535	5535.566	**5532.620**
	Worst	10919.04	8724.383	5591.429	5538.734	**5534.836**
	Std.	682.494	306.242	9.928	1.775	**0.423**
	F-measure	0.486	0.4875	0.517	0.487	**0.52107**
	Time (s)	**0.870**	2.098	86.887	72.470	59.485
Glass	Best	223.509	216.911	214.382	213.726	**210.17**
	Mean	245.893	221.685	215.877	216.187	**211.214**
	Worst	352.186	225.658	220.359	221.714	**213.686**
	Std.	27.720	3.002	1.266	1.814	**1.174**
	F-measure	0.589	0.611	0.597	0.611	**0.680**
	Time (s)	**0.079**	0.253	37.199	26.606	28.459
Cancer	Best	3022.224	2984.068	2965.945	2964.722	**2964.393**
	Mean	3342.213	2984.674	2973.296	2966.021	**2964.522**
	Worst	4316.204	2985.659	2994.217	2976.272	**2964.73**
	Std.	346.972	0.645	5.743	2.753	**0.091**
	F-measure	0.957	**0.967**	0.964	**0.967**	0.964
	Time (s)	**0.283**	0.802	23.607	16.8370	14.736

Furthermore, the trade-off between the quality and the time-cost problem occurred, leading to the time-cost-quality trade-off problem. The hybrid metaheuristic approaches, such as AMADE, HyDE, and HyGA, can obtain optimal solutions in reasonable execution time. In contrast, the traditional metaheuristic algorithm, such as GA and DE, do not guarantee to find the optimal solution, but they usually obtain sub-optimal, good-quality solutions in less execution time. As shown in Table 3, The traditional DE and DE algorithm achieve best execution time for all dataset, but they were unable to obtain the optimal solution for the datasets. In contrast, AMADE algorithm produced the optimal results of the intra-clusters distances and the F-measure with reasonable execution time when compared to HyDE and HyGA. For example, AMADE obtained 5532.620 for the average intra-cluster distance on CMC dataset, and 0.52107 for the F-measure in 59.485 seconds, which were the optimal results with the best execution time compared to HyDE (72.470) and HyGA (86.887).

**Fig 14:**
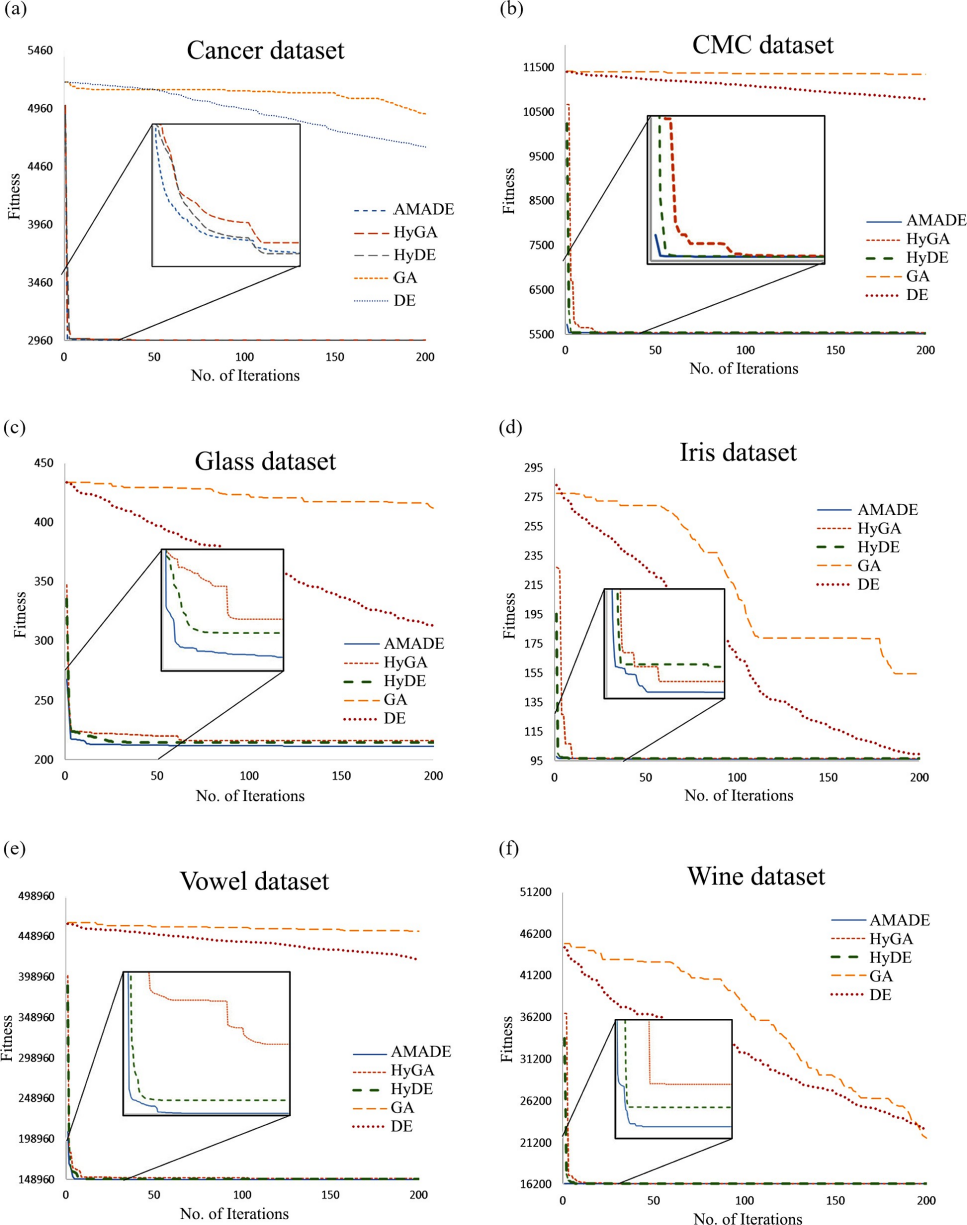
**The convergence curves for the first 200 iterations on (a) cancer, (b) CMC; (c) glass; (d) iris; (e) vowel; (f) wine datasets**.

Fig 14 shows the convergence curves of the first 200 iterations on six datasets. It demonstrates that AMADE has the best convergence rate results on the six datasets with faster converge in the early iterations of the search process; later, the convergence becomes slower. The HyDE achieved the second best convergence rate results, and the HyGA scored third best results. The GA and DE algorithm produced a slow convergence rate toward the optimum intra-cluster distance on all datasets. In general, the improved memetic phases by removing the duplicated solutions along with the local search and the adaptive strategy shown the effectiveness in preventing the algorithm from falling into premature convergence.

Furthermore, Table 4 shows the result of the rankings of the mean values generated by Friedman’s test based on the average and best value of intra-clusters distances. Additionally, the Friedman’s test reveals the significance of the AMADE algorithm with a *p*-value of 0. 000189 for the test based on the average value of intra-clusters distances, and 0.0000128 for the test based on the best value of intra-clusters distances, which are both below the significance level (α = 0.05).

**Table 4:** Friedman tests based on the average and best intra-clusters distances obtained by AMADE.

#	Algorithm	Ranking (Based on Average)	Ranking (Based on Best)
1	AMADE	1.00	1.00
2	HyGA	2.50	2.916
3	HyDE	2.50	2.083
4	DE	4.17	4.166
5	GA	4.83	4.833

The Holm’s procedure is employed as a post-hoc method to detect the statistical difference between the control case (ranked first) and the other remaining cases [72]. Table 5 shows the *p*-value obtained by the Holm’s procedure, where the rejection of the null hypothesis relies on the obtained *p*-value. Thus, the *p*-value must be less than the adjusted value of α (α*/i*), where *i* is the rank of the algorithm. Table 5 presents the adjusted *p*-value of Holm’s procedure, and the AMADE algorithm is used as the control algorithm. Holm’s procedure proves that AMADE is statistically better than DE, GA and HyGA, but the algorithm does not differ significantly from the HyDE algorithm. However, the results reported in Table 5 demonstrate that the proposed AMADE approach outperformed the HyDE in all of the tested datasets in all criteria. Based on the standard deviation criterion, AMADE is considered and more robust than HyDE as well as the other algorithms. Moreover, AMADE can found global optimal solutions for most of the cases.

**Table 5:** Holm’s procedure Adjusted p-value of the methods in the comparison.

i	Algorithm	α/i	p-value of Holms (based on average)	p-value of Holms (based on best)	Null Hypothesis
1	HyGA	0.05/1 = 0.0500	0.1003	0.03576	Not rejected, rejected
2	HyDE	0.05/2 = 0.0250	0.1003	0.2353	Not rejected, Not rejected
3	DE	0.05/3 = 0.0166	0.00052	0.000522	Rejected, rejected
4	GA	0.05/4 = 0.0125	0.000267	0.000026	Rejected, rejected

**Fig 15:**
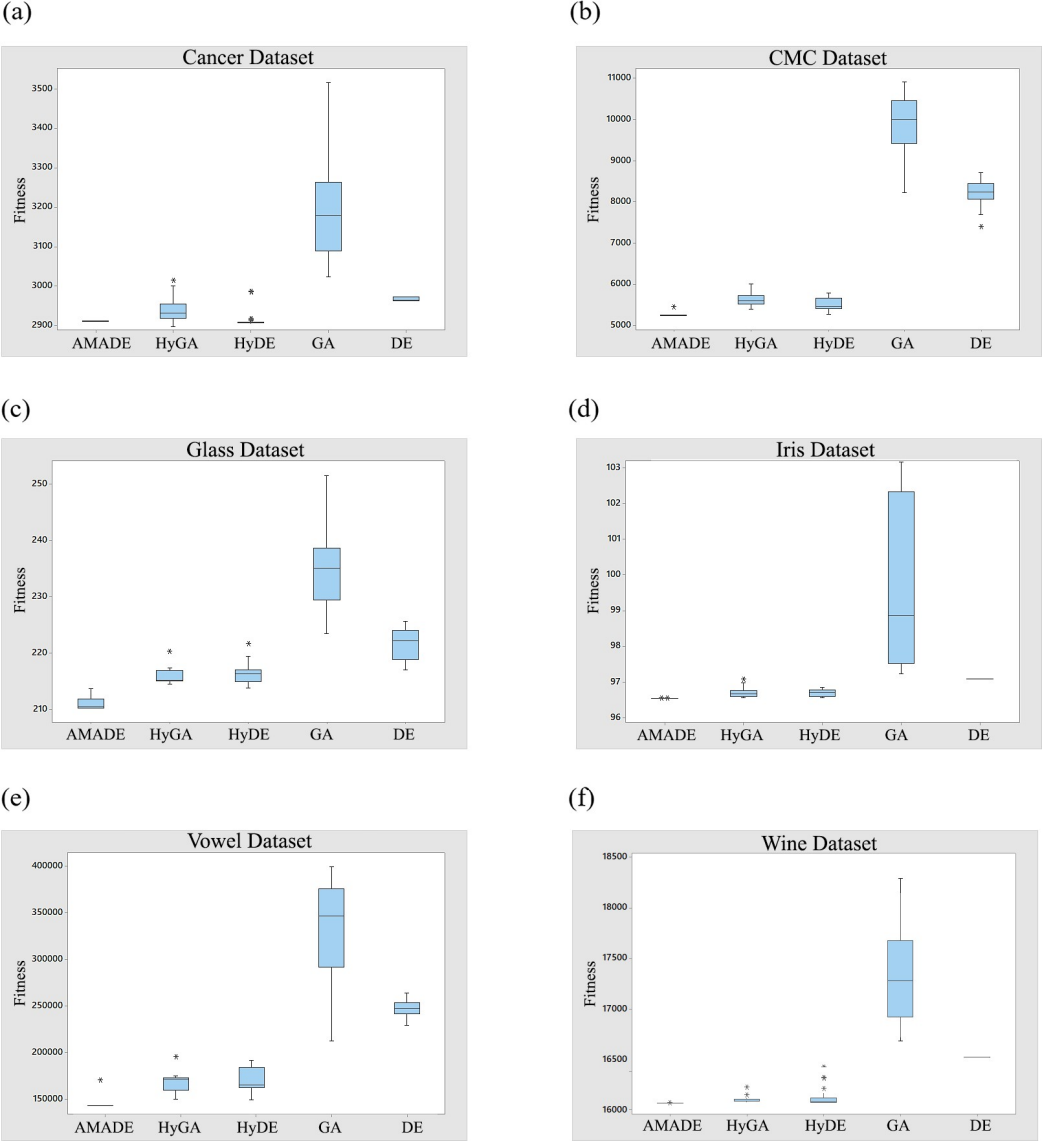
**Box plots of fitness of best solutions for AMADE, HyGA, HyDE, GA and DE algorithms on (a) cancer; (b) CMC; (c) glass; (d) iris; (e) vowel; (f) wine datasets**.

Additionally, in order to show the superiority of the AMADE algorithm among the other algorithms, Fig 15 presents the box plots of all datasets from 31 runs. It reveals that AMADE did not produce any outlier on all datasets, and the median solutions obtained by AMADE distributions are centralised. The box plots for the AMADE was thick and near the minimum intra-clusters distance values. The thickness of the box plots indicates that results obtained have less deviation of the median value, which means that the algorithm performance was stable over the 31 runs. The HyDE algorithm achieved the second best performance on Cancer, CMC, and Iris datasets, while it almost obtained the same performance of the HyGA algorithm on the Glass, Vowel, and Wine datasets. The standard DE algorithm obtained a better result than the GA algorithm on all datasets, where both GA and DE performance are weak compared with other hybrid algorithms. In general, the improved memetic phases by the restart phase along with the DE mutation phase shown the effectiveness in keeping the diversity of the population as maximum as possible during the evolutionary process, which helped to avoid the instability of the obtained results.

Furthermore, in order to validate the feasibility of the results, the centres of the clusters obtained by AMADE algorithm is shown in Tables 6–8, where all datasets with the same number of clusters are grouped in one table. The clusters centres can be used to validate the sum of intra-cluster distances given in Table 3. This could be manipulated by assigning the data objects within each dataset with the nearest clusters centres given accordingly in Tables 6–8, where the best intra-clusters distance values in Table 3 must be reached. For example, by allocating the 178 data objects in Wine dataset to the nearest centres with corresponding three cluster centres that are shown in Table 6, the best value of the sum of intra-cluster distances obtained by the AMADE algorithm on the Wine dataset, which is reported in Table 3, should be equals (16292.279). Otherwise, the best centres in Table 6 or the best values in Table 3 is invalid. This procedure can also be performed to validate other dataset’s cluster centres.

**Table 6:** The best clusters centres on the datasets Wine, Iris, and CMC obtained by the AMADE algorithm.

Dataset	Centre 1	Centre 2	Centre 3
Iris	6.731	5.019	5.93
	3.072	3.423	2.797
	5.629	1.469	4.416
	2.107	0.238	1.417
CMC	33.487	24.409	43.63
	3.137	3.04	3.01
	3.559	3.512	3.458
	3.653	1.798	4.595
	0.813	0.933	0.797
	0.707	0.817	0.764
	2.11	2.303	1.848
	3.293	2.967	3.438
	0.073	0.043	0.113
Wine	12.526	13.718	12.832
	2.389	1.861	2.582
	2.329	2.424	2.393
	21.391	16.905	19.509
	92.435	105.146	98.835
	2.09	2.76	2.088
	1.863	2.846	1.51
	0.408	0.293	0.429
	1.464	1.895	1.433
	4.349	5.601	5.801
	0.967	1.072	0.898
	2.525	3.015	2.258
	463.871	1137.495	686.798

**Table 7:** The best clustering centres on the Cancer data set obtained by the AMADE algorithm.

Dataset	Centre 1	Centre 2
Cancer	2.886	7.113
	1.127	6.639
	1.201	6.624
	1.165	5.613
	1.993	5.227
	1.122	8.097
	2.008	6.078
	1.099	6.021
	1.033	2.32
	2.886	7.113
	1.127	6.639
	1.201	6.624
	1.165	5.613

**Table 8:** The best clusters centres on the Vowel and Glass datasets obtained by the AMADE algorithm.

Dataset	Centre 1	Centre 2	Centre 3	Centre 4	Centre 5	Centre 6
Vowel	506.853	439.01	623.906	375.474	407.997	357.254
	1839.705	987.21	1309.831	2149.202	1018.042	2291.144
	2556.051	2665.001	2333.476	2678.136	2317.763	2977.367
Glass	1.52	1.517	1.513	1.517	1.522	1.521
	12.843	14.612	13.021	13.324	13.812	13.088
	3.456	0.056	0.018	3.578	3.569	0.259
	1.312	2.204	3.03	1.418	0.935	1.426
	73.022	73.239	70.581	72.669	71.854	72.666
	0.602	0.089	6.221	0.574	0.165	0.342
	8.565	8.651	6.94	8.212	9.523	11.978
	0.013	1.03	0.01	0.006	0.052	0.108
	0.079	0.016	0.001	0.05	0.058	0.061

### Comparison between AMADE and state of the art

In order to evaluate the performance of AMADE, the algorithm results are compared with well-known algorithms, such as the black hole (BH) [40], age-based particle swarm optimization (PSOAG) [68], A dynamic shuffled differential evolution algorithm (DSDE) [42], the krill herd algorithm (IKHCA) [69], hybrid of krill herd algorithm with harmony search algorithm (H-KHA) [17] and hybrid ICMPKHM [19].

The related comparison results are presented in Table 9. The results present the average of the intra-clusters distances for the AMADE and other Algorithms on Iris, Wine, CMC, Glass, and Cancer. The results indicate that AMADE has shown consistent performance and better result than IKHCA, ICMPKHM, PSOAG, H-KHA and BH on almost all the datasets. The AMADE achieved the second best results after the MSDE algorithm on Wine, CMC, Cancer datasets. Thus, The AMADE algorithm obtained the second best results on Iris and Glass datasets. The results shown in Table 9 reveal that the AMADE performance is consistent across all the datasets compared to the state of art algorithms concerning the average of the intra-clusters distances.

**Table 9:** Comparison between AMADE and other Algorithms based on the average of the intra-clusters distances.

Dataset	PSOAG	BH	DSDE	IKHCA	ICMPKHM	H-KHA	AMADE
Iris	96.97	96.65	96.65	96.67	96.61	**96.52**	96.549
Wine	16296.3	16294.3	**16292.3**	16589.	16293.18	16410.1	16292.8
CMC	5559.98	5533.63	**5532.18**	5695.0	5695.13	5601.68	5532.62
Glass	244.99	211.49	212.73	223.03	**199.45**	215.66	211.21
Cancer	2984.24	2964.39	**2964.38**	2971.1	3024.79	2982.43	2964.52

To further analyse the results in Table 9, the rankings with the compared algorithms generated by Friedman’s test are shown in Table 10 based on the average function of the intra-clusters distances. Furthermore, the Friedman’s test has shown a significant difference of the AMADE among the other compared algorithms, with a *p*-value of 0.02465 based on the average function, which is below the significance level (α = 0.05). The AMADE algorithm shares the best ranked algorithm with the DSDE algorithm [42], which uses the DE algorithm with multiple population approaches to reach the best average function of the intra-clusters distances for the best solutions. The results show that AMADE achieved the best ranking among other clustering algorithm based on the average performance function of the intra-clusters distances. The BH algorithm achieved the third best rank, and the ICMPKHM algorithm achieved the fourth rank, then the H-KHA. Lastly, PSOAG and IKHCA achieved the worst rank compared to other algorithms. The rankings generated by Friedman’s test shown in Table 9 reveal that the AMADE performance is consistent compared to the state of art algorithms concerning the average of the intra-clusters distances.

**Table 10:** Friedman tests based on the average of the intra-clusters distances.

Algorithm	Ranking
AMADE	2.2
DSDE	2.2
BH	3.4
ICMPKHM	4.2
H-KHA	4.4
PSOAG	5.8
IKHCA	5.8

Furthermore, the performance of AMADE is compared based on the computed accuracy with four algorithms that reported accuracy performance measure in their research, such as PSOAG, K-means [73], PSOAG, DSDE and IKHCA as shown in Table 11. The accuracy obtained by AMADE is competitive with the other clustering algorithm, where it reaches the optimum accuracy on CMC and cancer datasets. The IKHCA algorithm achieved best results of the accuracy on Wine, CMC, and Glass datasets, while the PSOAG algorithm achieved the best result on the Iris dataset. However, the results of the accuracy reveal the consistent performance of the AMADE algorithm based on the accuracy on all datasets, where it obtained second best result of accuracy on Glass and Wine datasets and obtained the third best result of the accuracy on the Iris dataset.

**Table 11:** Comparison between AMADE and other population-based algorithms based on accuracy.

Data Set	K-means	PSOAG	DSDE	IKHCA	AMADE
Iris	83.3	**91.03**	90.00	90.67	90.0
Wine	63.62	70.98	71.65	**73.03**	71.9
CMC	41.8	39.87	38.49	**45.62**	**45.62**
Glass	60.8	51.26	53.48	**65.88**	63.08
Cancer	93.37	96.31	**96.486**	95.16	**96.486**

At last but not least, the performance of AMADE is compared based on the computed F-measure with three algorithms that have reported the F-measure external performance measure in their research, such as K-means [74], KSC-LCA [74], ICMPKHM [19] as shown in Table 12. The F-measure obtained by AMADE outperformed other clustering algorithms, where it reached the optimum F-measure value on the Iris, CMC, Cancer and Vowel datasets, while it obtained the second best results of the F-measure on Wine and Glass. The KSC-LCA algorithm achieved the best result of the F-measure on Wine dataset, and ICMPKHM algorithm achieved the best result on Glass and Cancer datasets. The results shown in Table 12 reveals the consistent performance of AMADE across all dataset based on the F-measure.

**Table 12:** Comparison between AMADE and other population-based algorithms based on the F-measure.

Data Set	K-means	KSC-LCA	ICMPKHM	AMADE
Iris	80.4572	89.8775	89.232	**90.1**
Wine	66.9781	**73.0221**	68.81	70.8
CMC	36.9273	42.8981	47.51	**52.10**
Glass	45.9440	49.6733	**69.52**	68.0
Cancer	95.6863	96.1730	**96.4**	**96.4**
Vowel	48.6859	51.9360	65.8	**66.20**

## Conclusions and future work

In this work, an adaptive memetic differential evolution (AMADE) was proposed for efficient data clustering. The combination between MA and DE algorithms aimed to balance between the exploration and exploitation. The algorithm proposed an adaptive DE mutation operator and a neighbourhood selection heuristic that are combined with memetic algorithm evolutionary steps. The enhancements helped to avoid the instability of the obtained results by keeping the diversity of the population as maximum as possible during the evolutionary process. Experiments conducted on six real-life datasets with different level of complexity have demonstrated that the AMADE showed consistent performance compared to the state of art algorithms concerning the average of the intra-clusters distances, accuracy, and F-measure validity measures. AMADE algorithm achieved the optimum result of the accuracy on CMC (45.62%) and Cancer (96.486%) datasets, and also reached the optimum result of the F-measure on Iris (90.1%), CMC (52.10%), Cancer (96.4%), and Vowel (66.20%) datasets. Moreover, future work will focus on using other data clustering objective functions to solve a variety of categorical and mixed data datasets. Additionally, future work will focus on how to associate validity measures with each other when combined in multi-objective approaches.

## Abbreviations

AMADE: Adaptive memetic differential evolution algorithm
BH: Black hole algorithm
DE: Differential Evolution algorithm
DSDE: Dynamic shuffled differential evolution algorithm
GA: Genetic Algorithm
GSA: Gravitational search algorithm
H-KHA: Hybrid of KH with HS algorithms
HS: Harmony search algorithm
ICA: Imperialist Competitive Algorithm
ICMPKHM: Combined KHM with ICS and PSO algorithms
ICS: Improved Cuckoo Search
KH: Krill herd algorithm
KHM: K-harmonic means algorithm
MA: Memetic Algorithm
PSO: Particle Swarm Optimizer
PSOAG: Age-based particle swarm optimization algorithm
|R_i_|: The number of objects in class R_i_
|S_j_|: The number of data objects in cluster S_j_
CandPoolSize: Recombination mating pool size
C_best_: Best individual centroid
C_current_: The current individual centroid
Cr: Crossover constant
C_rand_: Random individual centroid
d(O_i_, O_j_): The Euclidean distance between objects O_i_ and O_j_
d(O_i_, Z_l_): The Euclidean Distance between the centre of cluster Z_l_ and object O_i_
F: DE Differentiation constant
f(X, C): Fitness function to measure the quality of the partitions
K: Number of clusters in the dataset
MGWI: Max generation without improvement constant
n_l_: Number of data objects in cluster Z_l_
PopSize: The population size constant
TourSize: Tournament selection size
